# Krüppel-like factors play essential roles in regulating pluripotency and the formation of neural crest stem cells

**DOI:** 10.1242/dev.204634

**Published:** 2025-05-01

**Authors:** Sara Rigney, Joshua R. York, Carole LaBonne

**Affiliations:** ^1^ Department of Molecular Biosciences, Northwestern University, Evanston, IL 60208, USA; ^2^ NSF-Simons National Institute for Theory and Mathematics in Biology, Northwestern University, Evanston, IL 60208, USA

**Keywords:** Neural plate border, Neural crest, *klf17*, *klf2*, *Xenopus*, Lamprey

## Abstract

The evolution of complex vertebrate body plans was driven by the acquisition of the neural crest, a stem cell population that retains broad, multi-germ layer potential after most embryonic cells have become lineage restricted. We have previously shown that neural crest cells share significant gene regulatory architecture with pluripotent blastula stem cells. Here, we examine the roles that two Krüppel-like Family (Klf) transcription factors, Klf2 and Klf17, play in these cell populations. We found that inhibition of either *klf2* or *klf17* expanded expression of pluripotency, neural plate border and neural crest factors in neurula stage *Xenopus* embryos, suggesting that Klf factors regulate the exit from pluripotency and proper establishment of the boundary of the neural crest domain. To gain further insights into the role of Klf factors in the evolution of the neural crest, we examined their expression in sea lamprey, a jawless vertebrate, and show that ectopic expression of lamprey *klf17* in *Xenopus* embryos phenocopies *Xenopus klf17*. These data suggest that *klf17* may have been the ancestral Klf factor that functioned in these gene regulatory networks in stem vertebrates.

## Teaser

**Summary:** Klf2 and Klf17 control the exit of blastula stem cells from pluripotency and restrict the boundaries of the neural plate border and neural crest domains in *Xenopus*.

## INTRODUCTION

The neural crest is a stem cell population, unique to vertebrates, that is notable for its broad multi-germ layer developmental potential (Dupin and Le Douarin, 2014; Le Douarin and Kalcheim, 1999; Prasad et al., 2012). Neural crest cells contribute an amazingly diverse array of highly specialized cell types to the vertebrate body plan, including sensory neurons and glia of the peripheral nervous system, pigment-producing melanocytes, and bone and cartilage of the craniofacial skeleton (Hall, 1999; Le Douarin and Kalcheim, 1999; Schock et al., 2023). Notably, these cells retain their broad developmental potential even as neighboring cells become lineage restricted. Thus, understanding the developmental origins of neural crest stem cells and their broad potential is key to understanding the origin and maintenance of stem cell potential and the early evolution of vertebrates.

New insights into the origins of neural crest potential came from the realization that these cells share significant gene regulatory network (GRN) architecture with the pluripotent cells of vertebrate blastula-stage embryos, including a large cohort of transcription factors (Buitrago-Delgado et al., 2015; Lavial et al., 2007; Lignell et al., 2017; Lukoseviciute et al., 2018; Pajanoja et al., 2023; Scerbo and Monsoro-Burq, 2020; Zalc et al., 2021; York et al., 2024). These findings suggest a model where neural crest cells arose via retention of characteristics of those earlier cells. Consistent with such a model, a requirement for BMP signaling, and FGF-mediated MAP kinase signaling have also been found to be shared attributes of pluripotent blastula cells and neural crest cells in *Xenopus* (Geary and LaBonne, 2018; Nordin and LaBonne, 2014). Both stem cell populations are also characterized by low levels of histone acetylation and a requirement for both histone deacetylase (HDAC) and Brd4 activity, indicating the important roles that epigenetic factors play in these cells (Rao and LaBonne, 2018; Huber et al., 2024).

Recent comparative genomic work in *Xenopus* and lamprey has demonstrated that a shared pluripotency-neural crest GRN had already been assembled in stem vertebrates (York et al., 2024), providing further evidence that the multi-germ layer potential of the neural crest evolved in early vertebrates by deploying this shared regulatory program. Lampreys, one of only two extant jawless vertebrates (the other being hagfish) have evolved independently from jawed vertebrates for more than 500 million years (Miyashita et al., 2019; Smith et al., 2018). Thus, traits shared between lampreys and jawed vertebrates such as *Xenopus* likely represent those present in the last common ancestor of extant vertebrates. Notably, both the blastula and neural crest GRNs were found to be highly conserved between *Xenopus* and lamprey, even at the level of absolute transcript levels (York et al., 2024). There were, however, a number of notable differences between the lamprey and *Xenopus* GRNs. One of these centered on Krüppel-like factors (Klfs), a large diverse family of transcription factors characterized by three highly conserved zinc fingers in their C-terminal DNA-binding domains (Miller and Bieker, 1993; Pearson et al., 2008; Presnell et al., 2015). These factors can act as either transcriptional activators or repressors (Jha et al., 2024). In mammals, Klf2 and Klf4 have established roles in pluripotency in blastula embryos and derived embryonic stem cells (Bieker, 2001; Jiang et al., 2008; Bourillot and Savatier, 2010; McConnell and Yang, 2010; Yeo et al., 2014; Bialkowska et al., 2017; Lea et al., 2021). Unlike mammals and other amniotes, however, lamprey Klf2/4 is not expressed in blastula or neural crest stem cells (Hockman et al., 2019; York et al., 2024), whereas a closely related factor, Klf17, is.

Klf4 is one of the original Yamanaka factors, a transcription factor that, in combination with Myc, Sox2 and Oct4 (Pou5f), was shown capable of reverting differentiated somatic cells back to a pluripotent state in mammalian cell culture (Takahashi and Yamanaka, 2006; Park et al., 2024). Follow-up studies expanded the network of proteins underpinning pluripotency to include Sall4, Foxd3 and *gbx2*, as well as Zic and Tfap2 family transcription factors (Hanna et al., 2002; Lim et al., 2010; Swaidan et al., 2020; Wang et al., 2017; Xiao et al., 2024). Moreover, with respect to reprogramming somatic cells, it was found that Klf2 could replace Klf4 (Nakagawa et al., 2008; Feng et al., 2009; Gillich et al., 2012). Indeed, a Klf regulatory network consisting of Klf2, Klf4 and Klf5 is involved in maintaining pluripotency in mouse embryonic stem cells (mESCs) (Jiang et al., 2008; Parisi et al., 2008; Yeo et al., 2014). It has been reported that, due to at least partial functional redundancy, knockdown of all three Klf factors is required to induce the differentiation of mESCs (Jiang et al., 2008). Notably, all three Klf factors share key regulatory targets with the pluripotency factor Nanog, including *Pou5f1* and *Sox2* (Jiang et al., 2008; Parisi et al., 2008; Bourillot et al., 2009; Hall et al., 2009; Wei et al., 2009; Zhang et al., 2010; Yeo et al., 2014). Moreover, all three of these Klf factors bind upstream regulatory regions in the *Nanog* promotor and promote its expression (Jiang et al., 2008). Despite these redundancies, genome occupancy experiments indicate that each of these Klf factors also regulates unique targets, and suggest that they function hierarchically to promote pluripotency (Jiang et al., 2008; Parisi et al., 2008; Yeo et al., 2014). Despite their well-studied and crucial role in regulating pluripotency in cultured mESCs, the role of Klf factors in regulating pluripotency in blastula stem cells *in vivo*, as well as their role in neural crest cells during development, has remained largely unexplored.

Here, we show that of the three Klfs required in mESCs, only *klf2* is highly expressed in the blastula stem cells of *Xenopus* embryos. We further show that *Xenopus* blastula stem cells express low levels of a related factor, *klf17*, which is subsequently expressed at higher levels in the neural plate border and neural crest. We provide evidence that Klf2 and Klf17 regulate the exit from pluripotency in early embryonic cells and subsequently control the expression boundaries of neural plate border and neural crest genes. Despite the temporal expression differences of *klf2* and *klf17*, our functional studies show that the proteins they encode have largely overlapping activities. Finally, we explore the evolutionary origins of Klf protein activity in blastula and neural crest stem cells. We provide evidence for deep conservation of Klf17 function in both stem cell populations across cyclostomes and gnathostomes and our data suggest that Klf17, rather than Klf2/4, was likely the ancestral Klf factor involved in regulating the developmental potential of blastula and neural crest stem cells in early vertebrates.

## RESULTS

### *klf2* and *klf17* are expressed in blastula and neural crest stem cells

**Fig. 1.**
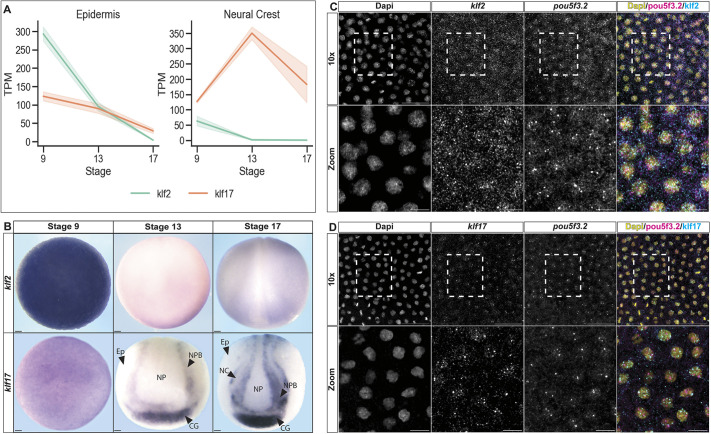
***klf2* and *klf17* are dynamically expressed in the blastula, neural plate border and neural crest cells of *Xenopus* explants and embryos.** (A) Graph depicting average TPMs of *klf2* and *klf17* in epidermis and neural crest explants at stages equivalent to blastula (stage 9), early neurula (stage 13) and late neurula (stage 17) of whole embryos. Data are mean±s.e.m. (B) *In situ* hybridization examining *klf2* and *klf17* expression in whole-mount wild-type *Xenopus* embryos collected at blastula (stage 9), early neurula (stage 13) and late neurula (stage 17) stages. Scale bars: 100 µm. (C,D) Blastula animal pole explants probed with HCR oligos examining the expression of the pluripotency factor *pou5f3.2* (magenta) with (C) *klf2* or (D) *klf17.* The area outlined in the top rows is shown in more detail in the bottom row. Scale bars: 150 µm (top); 75 µm (bottom)*.* NP, neural plate; NPB, neural plate border; NC, neural crest; CG, cement gland; Ep, epidermis.

To determine which Klf family genes are expressed in blastula and/or neural crest stem cells in *Xenopus* embryos, we mined our previously published transcriptomes for pluripotent blastula and neural crest stem cells (York et al., 2024) for all known Klf factors in the *Xenopus* genome. In contrast to mESCs (Jiang et al., 2008; Parisi et al., 2008; Yeo et al., 2014) we found that *Xenopus klf2* was the most highly expressed Klf factor in blastula animal pole cells (Fig. S1A), and its expression was approximately 17-fold higher than that of *klf4*. When we examined the expression of Klf factors in blastula cells that had been induced to adopt a neural crest state, we found that *klf17* (previously called Neptune; Kurauchi et al., 2010) was the only family member whose expression increased in early (stage 13) neural crest cells. Additionally, it was the most highly expressed Klf factor in both stage 13 and stage 17 neural crest cells (Fig. S1A). By contrast, *klf2* expression was reduced in neural crest cells relative to its blastula expression (Fig. 1A). While *klf17* was also expressed in blastula stem cells, its expression was significantly (2.4-fold; *P*=0.002) lower than that of *klf2*.

We next examined the temporo-spatial expression of *klf2* and *klf17* in *Xenopus* embryos using whole-mount *in situ* hybridization (WISH). Consistent with our transcriptomic data, we observed high expression of *klf2* in blastula animal pole cells and significantly lower expression of *klf17*. Both *klf2* and *klf17* colocalized with pluripotency maker *pou5f3.2* in these cells (Fig. 1C,D). At early neurula stages (stage 13), *klf17* was expressed at the neural plate border and the presumptive cement gland. *klf2* expression was undetectable by WISH at stage 13, although transcripts were detected by RNA-Seq. By stage 17, low levels of *klf2* expression were observed throughout the non-neural ectoderm. Interestingly, by this stage, *klf17* expression in the neural folds had resolved to distinct medial and lateral domains (Fig. 1B).

**Fig. 2.**
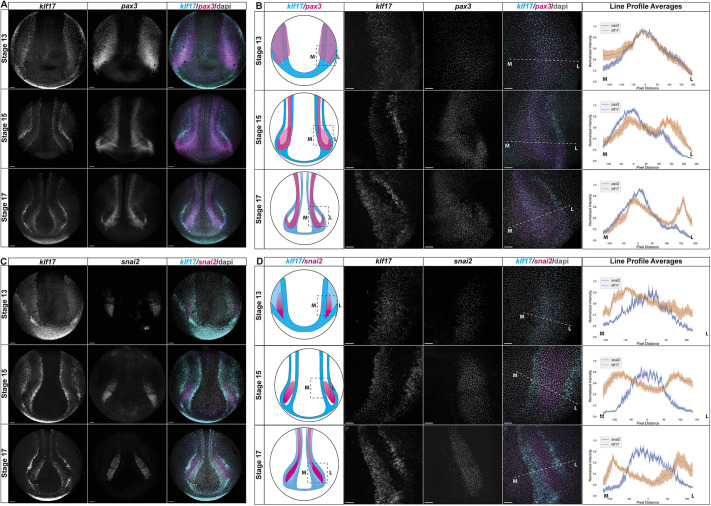
**The overlapping expression patterns of *klf17* with neural plate border and neural crest change during neurulation.** (A,C) Whole *Xenopus* embryos probed with HCR oligos examining the expression patterns of (A) *klf17* (cyan) and the neural plate border marker *pax3* (magenta), or (C) *klf17* (cyan) and the neural crest marker *snai2* (magenta) at early (stage 13), mid (stage 15) and late (stage 17) neurulation. (B,D) Average line profile measurements of the normalized pixel intensity values across the medial-lateral axis of maximum projection images of the neural plate border and neural crest cell regions of wild-type embryos. Diagrams in the first column represent the embryos and gene expression patterns at each neurula stage analyzed. The area of the embryo imaged for each line profile measurement is outlined. Representative individual (B) *klf17* and *pax3*, or (D) *klf17* and *snai2* HCR images are shown in the second and third columns, as well as the merged channel image in the fourth column. Dotted lines on the merged images depict the axis that the line profile averages were measured across. M and L indicate, respectively, the medial and lateral endpoint with respect to the embryo midline. The right-most column plots the average line profile measurements for each stage (minimum *n*=5). The *x*-axis shows pixel positions along the measurement axis, with the peak of either *pax3* (B) or *snail2* (D) profiles centered at 0 (after curve fitting). The *y*-axis shows the average normalized intensity values of either (B) *pax3* (blue) and *klf17* (orange), or (D) *snai2* (blue) and *klf17* (orange) with shading indicating the s.e.m. Scale bars: 150 µm.

We next used hybridization chain reaction (HCR) probes to further examine the expression of *klf17* at neurula stages and compare its expression to neural plate border and neural crest factors. We observed significant overlap of *klf17* and *pax3* expression at the neural plate border at stage 13 (Fig. 2A,B). While *snai2* expression was much lower at this stage, most *snai2-*positive cells also expressed *klf17* (Fig. 2C,D, Fig. S2D). Strikingly, however, by stage 17 the medial and lateral *klf17* expression domains surrounded the *snai2* domain but did not overlap with it. Cells within the medial band of *klf17* expression still co-expressed *pax3* at this stage (Fig. 2A,B). We used line profile averages of confocal images to further illustrate these changes that occur to the spatial relationships between *klf17* and *pax3*, and *klf17* and *snai2* expression domains during the establishment of the neural plate border and neural crest (Fig. 2B,D). We extended this characterization by using HCR to compare *klf17* expression to an additional neural plate border factor, *zic1*, an additional neural crest marker, *foxd3*, and the neural plate marker *sox2* (Fig. S2A-C). Again, we observed the most overlap in expression at stage13, and *klf17* expression surrounding *foxd3* at stage 17 (Fig. S2B).

### *klf2* or *klf17* depletion expands the neural plate border and neural crest domains

**Fig. 3.**
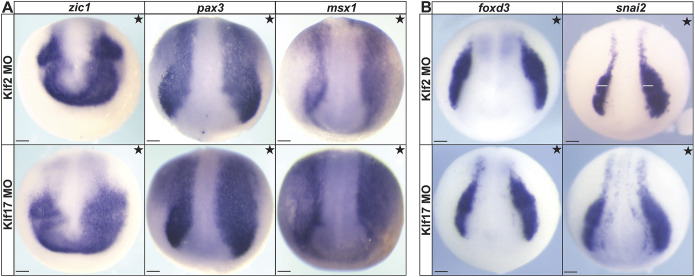
***klf2* and *klf17* are essential for establishing the proper neural plate border and neural crest domain boundaries.** (A,B) *In situ* hybridization of *Xenopus* embryos unilaterally injected with either *klf2* or *klf17* fluorescein-tagged morpholinos (stars indicate the injected side). The fluorescein tag was used as a lineage tracer and embryos were pre-sorted for left/right targeting. (A) Neural plate border markers *pax3*, *zic1* and *msx1* in early neurula embryos. (B) Neural crest cell markers *foxd3* and *snai2* in late neurula embryos. The white lines measure the width of control neural crest size. MO, morpholino. Scale bars: 100 µm.

Having characterized the expression of *klf2* and *klf17*, we next asked if these factors are required for establishment of the neural plate border and/or neural crest. To this end, we injected two cells of 8-cell embryos with translation-blocking morpholinos (MO) specific to each of these Klf factors (Fig. S3A) and cultured them to neurula stages for WISH. We found that MO-mediated depletion of either *klf2* or *klf17* resulted in expanded expression of the neural plate border markers *zic1* (Klf2MO: 90.5%, *n*=74; Klf17MO: 88.3%, *n*=77), *pax3* (Klf2MO: 86.2%, *n*=65; Klf17MO: 83.1%, *n*=83) and *msx1* (Klf2MO: 75%, *n*=60; Klf17MO: 85%, *n*=60) relative to the uninjected side (Fig. 3A). As neural plate boarder factors are required for the formation of definitive neural crest cells (Hong and Saint-Jeannet, 2007; Monsoro-Burq et al., 2005; Groves and LaBonne, 2014), we next examined the effects of *klf2* or *klf17* depletion on the expression of neural crest factors. Depletion of either *klf2* or *klf17* resulted in expanded expression of s*nai2* (Klf2MO: 77.1%, *n*=166; Klf17MO: 68.4%, *n*=177) and *foxd3* (Klf2MO: 70.8%, *n*=106; Klf17MO: 73.5%, *n*=136) (Fig. 3B), albeit less pronounced than that observed for neural plate border factors. Together, these findings indicate that, despite the temporal differences in their expression, both *klf2* and *klf17* are required for the proper establishment of the spatial boundaries of both the neural plate border and neural crest.

### Forced expression of *klf2* or *klf17* inhibits expression of neural crest factors

**Fig. 4.**
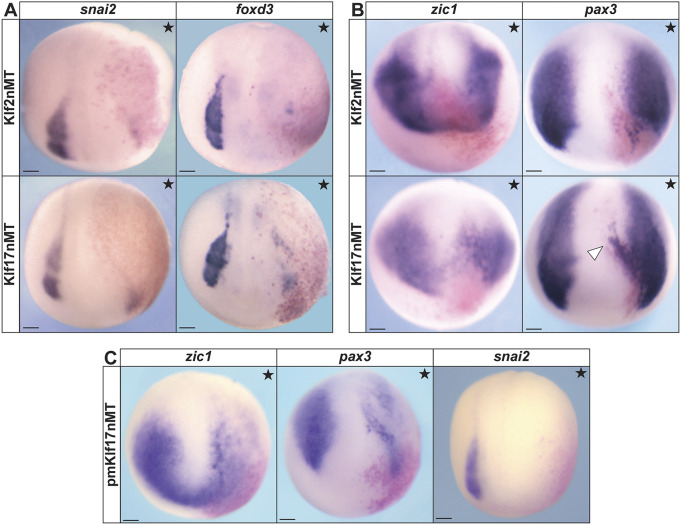
**Ectopic effects of Klf transcription factors on the establishment of proper neural plate border boundaries and neural crest cell formation are evolutionarily conserved.** (A,B) *In situ* hybridization of *Xenopus* embryos unilaterally expressing *klf2* or *klf17* epitope-tagged mRNA (stars indicate the injected side). β-Galactosidase (red) was used as a lineage tracer. (A) *snai2* and *foxd3* in late neurula embryos. (B) *pax3* and *zic1* in early neurula embryos. Arrowhead in B indicates ectopic neural plate expression. (C) *In situ* hybridization for *zic1*, *pax3* and *snai2* in neurula embryos unilaterally expressing *Petromyzon marinus* (sea lamprey) *klf17* epitope-tagged mRNA (stars indicate the injected side). β-Galactosidase (red) was used as a lineage tracer. nMT, *n*-terminal myc tag; pm, *Petromyzon marinus*. Scale bars: 100 µm.

Because the above loss-of-function experiments suggest that *klf2* and *klf17* act directly or indirectly to restrict the size of neural plate border and neural crest domains, we hypothesized that *klf2* and/or *klf17* gain of function might interfere with expression of neural plate border and/or neural crest factors. To test this, we injected one cell of two-cell stage embryos with mRNA encoding an N-terminal myc tag (nMT) *klf2* or *klf17* and cultured embryos to neurula stages for WISH. Western blot analysis was used to ensure proteins were expressed at equivalent levels (Fig. S4). Consistent with this hypothesis, ectopic activity of either *klf2* or *klf17* resulted in near-total loss of the neural crest factors *snai2* (Klf2nMT: 90.7%, *n*=107; Klf17nMT: 84.2%, *n*=114) and *foxd3* (Klf2nMT: 94.5%, *n*=91; Klf17nMT: 93.7%, *n*=95) (Fig. 4A). When effects on neural plate border markers *pax3* and *zic1* were examined, the phenotypes were more complex. Individual embryos expressing either Klf factor displayed regions of lost expression as well as regions of ectopic expression, primarily in the medial neural plate [*pax3* (Klf2nMT: 98.3, *n*=59; Klf17nMT: 91.7%, *n*=72) and *zic1* (Klf2nMT: 98.7%, *n*=75; Klf17nMT: 96.1%, *n*=102)] (Fig. 4B)*.* As both of these genes have a neural component to their expression, this may explain the ectopic expression observed in the neural plate. Together, these results suggest that both *klf2* and *klf17* regulate the spatial boundaries of the neural plate border and neural crest domains.

We have previously shown that, in lamprey, *klf17* is the only *klf2/4/17* clade member expressed in neural crest and blastula stem cells (York et al., 2024) (Fig. S5A). We therefore asked if the ability of *klf17* to restrict neural crest might be deeply conserved to the base of vertebrates by performing heterologous gain-of-function experiments. mRNA encoding epitope-tagged lamprey *klf17* was expressed unilaterally in two-cell stage *Xenopus* embryos at levels matching those of *Xenopus klf17*. We found that lamprey *klf17* largely phenocopied the repressive effects of *Xenopus klf17*, resulting in near-total loss of neural plate border and neural crest gene expression for *pax3* (100%, *n*=48), *zic1* (90.2%, *n*=41) and *snai2* (100%, *n*=61) (Fig. 4C). These results suggest that the ability of Klf17 transcription factors to inhibit neural plate border and neural crest has been largely conserved across jawed and jawless vertebrate lineages.

### *klf2* and *klf17* regulate expression of pluripotency associated genes

**Fig. 5.**
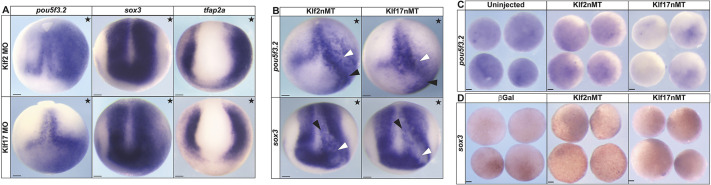
**Klf2 and Klf17 are essential for establishing the boundaries of pluripotency factors expressed in neurula-stage embryos.** (A) *In situ* hybridization for *pou5f3.2*, *sox3* and *tfap2a* in early neurula embryos unilaterally injected with either *klf2* or *klf17* fluorescein-tagged morpholinos (stars indicate the injected side). (B) *In situ* hybridization for *pou5f3.2* and *sox3* in embryos unilaterally expressing *klf2* or *klf17* epitope-tagged mRNA (stars indicate the injected side). White arrowheads indicate areas of loss; black arrowheads indicate ectopic expression. The fluorescein tag was used as a lineage tracer and embryos were pre-sorted for left- or right-side targeting. (C,D) *In situ* hybridization of either uninjected wild-type explants or explants expressing *klf2* or *klf17* epitope-tagged mRNA examining the expression of: (C) pouf5f3.2 (fluorescein dextran was used as a lineage tracer and explants were pre-screened for targeting); and (D) sox3 (β-galactosidase (red) was used as a lineage tracer). MO, morpholino; nMT, n-terminal myc tag. Scale bars: 100 µm.

Pluripotency genes are required for proper establishment of the neural plate border in *Xenopus* as components of the blastula stage pluripotency GRN, including *pou5f* and *soxb1* factors, are co-opted for establishment of this domain (Schock et al., 2024; York et al., 2024). We therefore examined the expression of pluripotency-associated genes during establishment of the neural plate border. We found loss of either *klf2* or *klf17* resulted in expanded expression of *pou5f3.2* (Klf2MO: 64.2%, *n*=53; Klf17MO: 81.3%, *n*=64), *sox3* (Klf2MO: 93.8%, *n*=48; Klf17MO: 91.5%, *n*=47) and *tfap2α* (Klf2MO: 95.5%, *n*=44; Klf17MO: 83.6%, *n*=55) at these stages (Fig. 5A), suggesting that *klf2* and *klf17* also play essential roles in determining boundaries of the pluripotency-associated gene expression domains as embryos progress from blastula to neurula stages. This expansion was particularly dramatic for *pou5f3.2* expression, perhaps indicative of a more-direct regulation of this target.

We also examined the effects of increased Klf activity on pluripotency markers (Fig. 5B). While we had hypothesized that Klf2 and Klf17 would inhibit the expression of markers such as *pou5f3.2* and *sox3*, the actual phenotype was more complex. Similar to their effects on the neural plate border markers *zic1* and *pax3*, both Klf2 and Klf17 partially inhibit the expression of these factors but also induced regions of ectopic expression with both phenotypes manifesting in individual embryos (*pou5f3.2*: Klf2nMT: 91.2%, *n*=57; Klf17nMT: 86.5%, *n*=52; *sox3*: Klf2nMT, 87.8%, *n*=49; Klf17nMT, 83.3%, *n*=48). Because components of the pluripotency GRN are known to have complex cross-regulatory interactions, it is likely that klf2/17 gain of function has distinct regional effects depending on what interacting factors are expressed there and at what level.

Given the complexity of the whole embryo phenotype in response to increased Klf2 and Klf17 activity we turned to explant assays, where the responding tissue would be more uniform, allowing us to more effectively assess the role of Klfs. Two-cell embryos were injected in both cells with mRNA encoding either epitope-tagged *klf2* or *klf17* and cultured to blastula stage when animal pole cells were explanted and further cultured to stage 13 for analysis. No ectopic expression of *pou5f3.2* or *sox3* was observed indicating that the Klf-mediated ectopic expression of these factors in whole embryos was indirect (Fig. 5C,D; Fig. S6).

### Klf2 and Klf17 inhibit lineage restriction

**Fig. 6.**
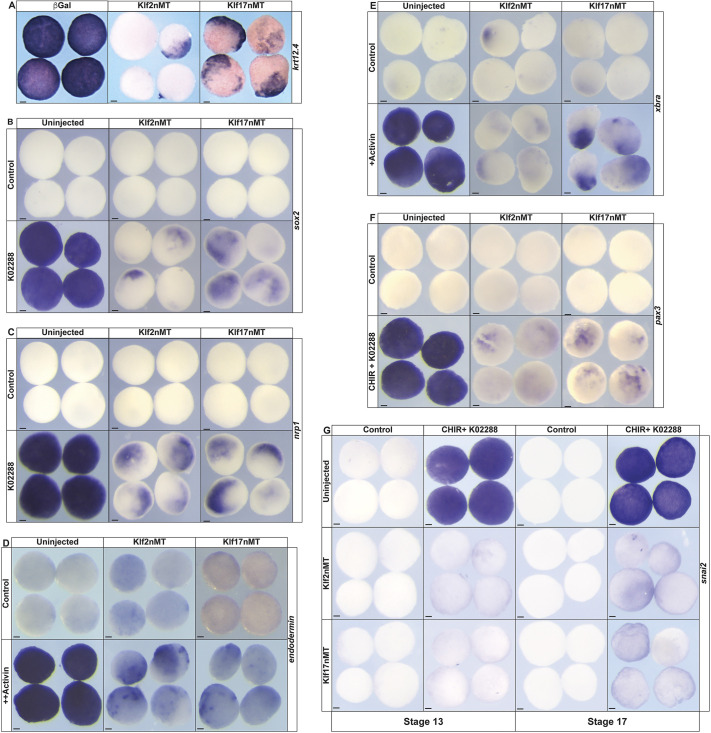
**Overexpression of *klf2* and *klf17* impairs lineage restriction.** (A-G) *In situ* hybridization of either uninjected wild-type explants or explants expressing *klf2* or *klf17* epitope-tagged mRNA examining the expression of: (A) epidermal marker *krt12.4* in untreated explants; (B,C) neural markers (B) *sox2* and (C) *nrp1* with or without the BMP antagonist K02288; (D) endodermal marker endodermin with or without a high dose of activin; (E) mesodermal marker *xbra* with or without a high dose of activin; (F) neural plate border marker *pax3* with or without a combination of a Wnt agonist (CHIR) and BMP antagonist (K02288) small-molecule cocktail; and (G) neural crest marker *snai2* with or without CHIR/K02288 cocktail at stages 13 and 17. nMT, n-terminal myc-tag; K02288, BMP inhibitor; CHIR, CHIR99021 (Wnt agonist); +, low activin; ++, high activin. Scale bars: 100 µm.

In *Xenopus* embryos, pluripotent blastula stem cells can easily be explanted and instructed to give rise to any cell type. Given no alternative signals, these cells transit to an epidermal stage due to autocrine BMP signaling, and will express the keratin *krt12.4*. To determine if upregulation of Klf2 or Klf17 activity would impact the ability of blastula cells to transit towards lineage restriction, two-cell embryos were injected in both cells with mRNA encoding either Klf2 or Klf17 and cultured to blastula stage when animal pole cells were explanted and further cultured to stage 13 for analysis. In contrast to uninjected control explants, explants expressing ectopic Klf2 or Klf17 failed to form epidermis, as evidenced by the loss of *krt12.4* (Klf2nMT: 81%, *n*=42; Klf17nMT: 87.2%, *n*=47; uninjected: 0%, *n*=34) (Fig. 6A; Fig. S6).

Treatment with a small molecule antagonist of BMP signaling, K02288, directs blastula explants to adopt a neural fate and express neural progenitor genes such as *sox2* (uninjected: 100%, *n*=34) and *nrp1* (uninjected: 100%, *n*=31) (Fig. 6B,C). Ectopic expression of either *klf2* or *klf17* prevented explants from adopting a neural fate with an observed loss of *sox2* (Klf2nMT: 96.9%, *n*=32; Klf17nMT: 90.3%, *n*=31) and *nrp1* (Klf2nMT: 100%, *n*=33; Klf17nMT: 93.8%, *n*=32) expression (Fig. 6B,C).

Explants can be induced to form endoderm by treatment with high doses of activin, as evidenced by expression of *endodermin*. Ectopic expression of either Klf2 or Klf17 blocked *endodermin* induction (Klf2nMT: 100%, *n*=32; Klf17nMT: 93.8%, *n*=32, uninjected: 0%, *n*=32) (Fig. 6D). Similarly, treatment with low doses of activin promote mesoderm formation as evidenced by *xbra* expression, and this too was blocked by either *klf2* or *klf17* (Klf2nMT: 90.9%, *n*=33; klf17nMT: 90%, *n*=30; uninjected: 0%, *n*=34) (Fig. 6E).

Finally, we asked whether these Klf factors would also inhibit neural crest or neural plate border formation in explants. Explants can be induced to a neural plate border or neural crest state by treatment with a combination of K02288 and the small molecule Wnt agonist CHIR99021 (CHIR) (Huber and LaBonne, 2024). Uninjected control explants treated with CHIR and K02288 strongly expressed the neural plate border factor *pax3* (100%, *n*=30), whereas ectopic expression of either *klf2* or *klf17* resulted in a failure to induce *pax3* expression (Klf2nMT: 91.4%, *n*=35; Klf17nMT: 97.2%, *n*=36) (Fig. 6F). We also examined the ability of Klf2 or Klf17 to block neural crest formation. We examined expression of *snai2* at both early and late neurula stages and found that it was strongly blocked at both stages [stage 13 (Klf2nMT: 100%, *n*=33; Klf17nMT: 100%, *n*=33, uninjected: 0%, *n*=42) and stage 17 (Klf2nMT: 96.8%, *n*=31; Klf17nMT: 100%, *n*=27, uninfected 0%, *n*=43)], indicating the onset of neural crest formation had been blocked (Fig. 6G). Together, these data support a model where forced expression of either Klf2 or Klf17 blocks the competence of blastula stem cells to respond to lineage-inducing cues.

### Loss of Klf activity prolongs expression of pluripotency factors in blastula explants

**Fig. 7.**
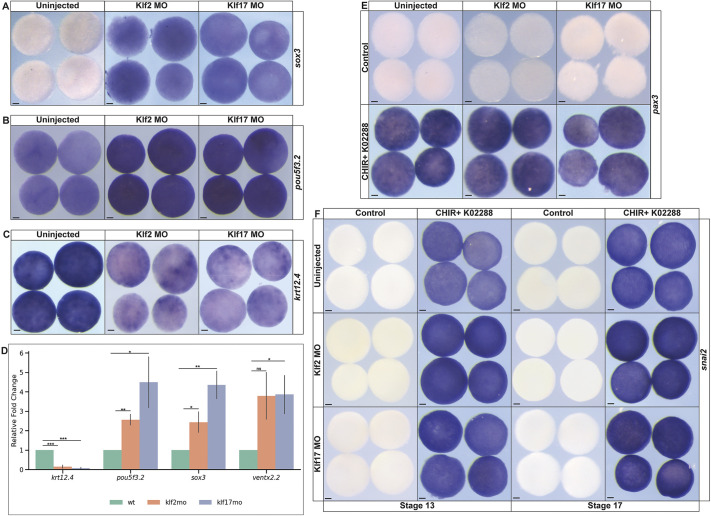
**Knockdown of *klf2* or *klf17* prolongs the expression of pluripotency factors.** (A-C) *In situ* hybridization of uninjected, *klf2* or *klf17* morphant explants (stage 12.5-13) examining the expression of: (A) *sox3*; (B) *pou5f3.2*; and (C) *krt12.4.* (D) qPCR of wild-type uninjected, *klf2* or *klf17* morphant explants (stage 12.5-13) for *krt12.4*, *pou5f3.2*, *sox3* and ventx2.2. *P≤0.05; **P≤0.01; ***P≤0.001 (unpaired, two-tailed *t*-test). Data are mean±s.e.m. (E,F) *In situ* hybridization of uninjected, Klf2 or Klf17 morphant explants of (E) *pax3* (stage 13) and (F) *snai2* (stages 13 and17) with or without the CHIR (Wnt agonist)+K02288 (BMP antagonist) small-molecule cocktail. MO, morpholino; K02288, BMP inhibitor; CHIR, CHIR99021 (Wnt agonist). Scale bars: 100 µm.

The above results demonstrate that Klf2 and Klf17 negatively regulate expression of neural crest and pluripotency factors, and block the competence of blastula stem cells to respond to inductive cues. We therefore hypothesized that loss of Klf2 and/or Klf17 might prolong the ability of pluripotent blastula cells to respond to inductive cues beyond stages when they are normally competent to do so. To test this, 8-cell embryos were injected in all four animal blastomeres with either *klf2* or *klf17* MOs, and animal pole cells were explanted at stage 9 and cultured to stage 13. While control explants had downregulated expression of *sox3* by this stage, explants depleted of *klf2* or *klf17* still expressed low levels of *sox3* (Klf2MO: 100%, *n*=34; Klf17MO: 100%, *n*=30; uninjected: 0%, *n*=31) (Fig. 7A). We also examined *pou5f3.2* and found that its expression was enhanced in response to either *klf2* or *klf17* depletion (Klf2MO: 93%, *n*= 43; Klf17MO 94.1%, *n*=34) (Fig. 7B). Consistent with the persistence of *sox3* and *pou5f3.2* expression, these explants did not properly lineage restrict, as evidenced by reduced expression of the epidermal marker *krt12.4* (Klf2MO: 93.5%, *n*=31; Klf17MO: 93.5%, *n*=31; uninjected: 0%, *n*=34) (Fig. 7C)*.* Interestingly, Klf depletion did not inhibit CHIR and K02288-mediated reprograming to a neural plate border state (Klf2MO: 93.3%, *n*=30; Klf17MO: 93.1%, *n*=29; uninjected: 100%, *n*=29) (Fig. 7E) likely because of the adjacency of this state to the pluripotent blastula state. Similarly, depletion did not impact the induction of the neural crest marker *snai2* [stage 13 (Klf2MO: 100%, *n*=42; Klf17MO, 100%, *n*=43, uninjected: 100%, *n*=42); stage 17(Klf2MO: 100%, *n*=46; Klf17MO: 100% *n*=42; uninjected: 100%, *n*=43)] (Fig. 7F).

### *klf2* and *klf17* regulate the exit from pluripotency

**Fig. 8.**
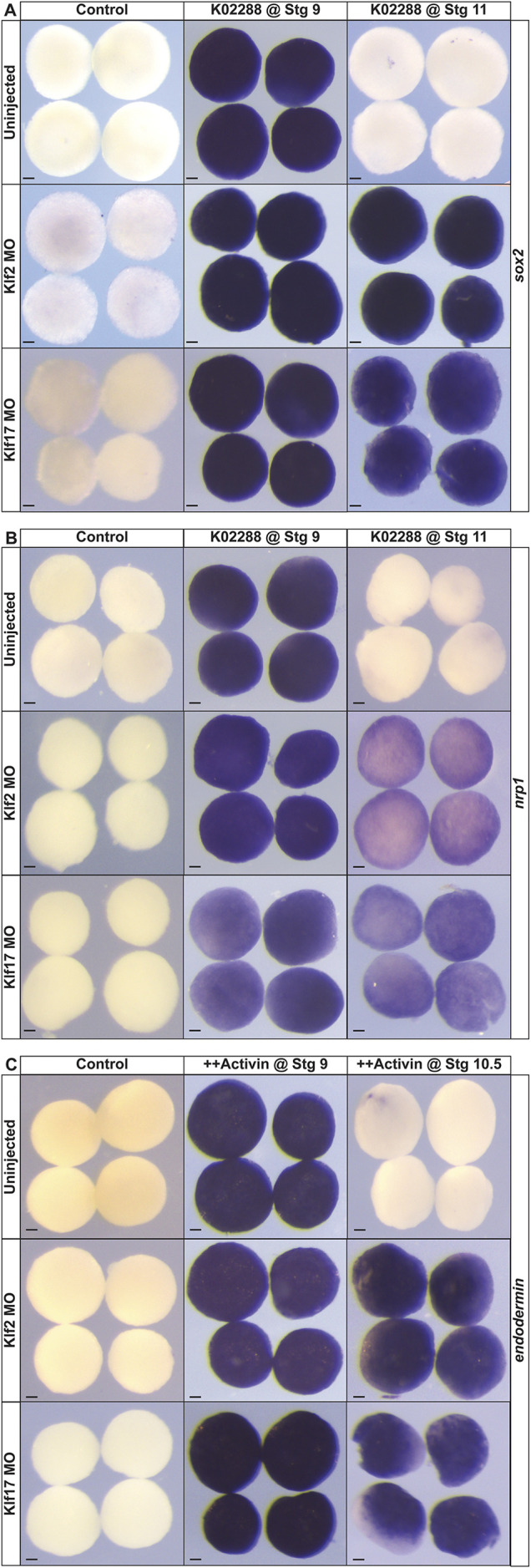
**Knockdown of *klf2* or *klf17* extends the window of competency to adopt different states.** (A-C) *In situ* hybridization examining the expression of neural markers (A) *sox2* and (B) *nrp1* in uninjected, *klf2* morphant or *klf17* morphant explants treated with the BMP antagonist K02288 at either stage 9 or stage 11 and then collected for analysis at stage 17. (C) *In situ* hybridization of examining the expression of endodermal marker endodermin in uninjected, *klf2* morphant or *klf17* morphant explants treated with high levels of activin at either stage 9 or stage 10.5, and then collected for analysis at stage 12. MO, morpholino; K02288, BMP inhibitor; CHIR, CHIR99021 (Wnt agonist); ++, high activin. Scale bars: 100 µm.

The expanded expression of pluripotency factors at the neural plate border and the prolonged expression of *pou5f3.2* and *sox3* in animal pole explants raised the possibility that loss of Klf2 or Klf17 was prolonging the pluripotency of blastula stem cells. We therefore asked if depletion of Klf2 or Klf17 would allow blastula explants to respond to neural-inducing cues beyond the time when control explants can do so. Treatment with the BMP inhibitor K02288 at stage 9 directs blastula explants to adopt a neural fate and express *sox2* or *nrp1* at stage 17 (Fig. 8A,B). Depletion of either Klf2 or Klf17 does not impede the induction of either *sox2* (Klf2MO: 97.8%, *n*=46; Klf17MO: 100%, *n*=45; uninjected: 100%, *n*=43) or *nrp1* (Klf2MO: 92.3%, *n*=39; Klf17MO: 95.7%, *n*=47; uninjected: 100%, *n*=40) expression when BMP signaling is inhibited at stage 9 (Fig. 8A,B). When BMP inhibition is initiated at stage 11.5, during gastrulation, cells are no longer competent to adopt a neural fate, as evidenced by a lack of *sox2* (97.1%, *n*=35) or *nrp1* (97.4%, *n*=38) expression. Strikingly, however, explants in which Klf2 or Klf17 have been depleted were able to adopt a neural fate when treated with K02288 at stage 9, as evidenced by expression of *sox2* (Klf2MO: 92.9%, *n*=42; Klf17MO: 95.1%, *n*=41) and *nrp1* (Klf2MO: 97.3%, *n*=37; Klf17MO: 97.2%, *n*=36) (Fig. 8A,B). This is consistent with a model whereby loss of *klf2* or *klf17* prolongs the pluripotency of blastula stem cells.

**Fig. 9.**
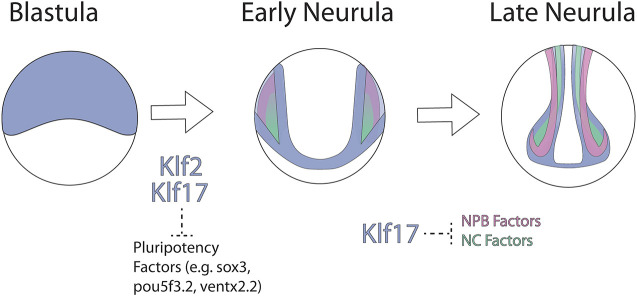
**Model for *klf2* and *klf17* function in blastula and neural crest stem cells.** Proposed model for Klf2- and Klf17-mediated regulation of pluripotency and neural plate border/neural crest gene expression. Klf2 and Klf17 (purple) are expressed in the blastula and regulate the exit from pluripotency. Klf17 but not Klf2 is expressed in the neural plate border (pink) and early neural crest cells (green), where it regulates expression of these genes through inhibitory mechanisms and sharpens the expression domain boundaries.

To determine if depletion of Klf2 or Klf17 prolonged pluripotency or only the competence to form neural progenitors, we next asked if loss of Klf2 or Klf17 could also prolong the ability of these explants to form endoderm. Treatment of control explants or explants depleted of *klf2* or *klf17* with high levels of activin at stage 9 induced strong expression of the endoderm marker *endodermin* at stage 13 (Klf2MO: 100%, *n*=26; Klf17MO:100%, *n*=30; uninjected: 100%, *n*=36) (Fig. 8C). Treatment of control explants with high concentrations of activin at stage 10.5 failed to induce endoderm, indicating that cells have lost the competence to respond by this stage. By contrast, explants depleted for either Klf2 or Klf17 were still competent to form endoderm in response to high activin at stage 10.5, as evidenced by strong *endodermin* expression (Klf2MO: 92.9%, *n*=28; Klf17MO: 93.5%, *n*=31, uninjected: 0%, *n*=34) (Fig. 8C). These data further support a model whereby Klf2 and *klf17* function to restrict rather than promote pluripotency (Fig. 9).

## DISCUSSION

Recent work has shown that there is a high degree of overlap between the GRNs of the neural crest and pluripotent blastula stem cells (Buitrago-Delgado et al., 2015; Zalc et al., 2021; Hovland et al., 2022; Pajanoja et al., 2023; Schock et al., 2023). At the core of the pluripotency GRN circuitry are the factors identified by Yamanaka [Pou5 (Oct3/4), Soxb1 (Sox2/3), Klf4, Myc] as sufficient to reprogram somatic cells to a pluripotent state (Takahashi and Yamanaka, 2006). These factors have been shown to be expressed in neural plate border and neural crest cells across multiple vertebrate species (Bellmeyer et al., 2003; Lavial et al., 2007; Buitrago-Delgado et al., 2015; Lignell et al., 2017; Bhattacharya et al., 2018; Scerbo and Monsoro-Burq, 2020; Zalc et al., 2021). Additionally, loss-of-function experiments have demonstrated that at least some of these key pluripotency factors are required for neural crest formation (Bellmeyer et al., 2003; Kurauchi et al., 2010; Scerbo and Monsoro-Burq, 2020; Zalc et al., 2021). Recent work in lamprey provided strong evidence that this shared pluripotency/neural crest GRN evolved at the base of the vertebrates (York et al., 2024).

A notable finding from that study was that, unlike mammals and other amniotes, lamprey *klf2/4* is not expressed in blastula stem cells or neural crest (York et al., 2024). Instead, it is *klf17* that is expressed in these stem cell populations. While *klf17* is also the most highly expressed Klf factor in *Xenopus* neural crest, it is *klf2* that is the predominant Klf factor expressed in pluripotent blastula cells. By contrast *klf4* is expressed in mouse neural crest cells (Pajanoja et al., 2023). Forced expression of Klf4 has been shown to prevent ES cell differentiation (Zhang et al., 2010), similar to what we find for Klf2 and Klf17. While Zhang et al. also proposed that Klf4 activity was required for both ES cell self-renewal and maintenance of pluripotency in mouse, more recent studies using inducible knockouts for Klf2, Klf4 and Klf5 have shown that all three must be knocked out to completely block self-renewal (Yamane et al., 2018). Moreover, expression of any of the three can rescue pluripotency. Interestingly, *KLF17* is expressed in the epiblast of pre-implantation human embryos (Blakeley et al., 2015), as well as in human naïve-like pluripotent stem cells (Takashima et al., 2014). As human pluripotent stem cells do not express *KLF2*, this would be consistent with a functional replacement by *KLF17* (Blakeley et al., 2015).

There is additional evidence, beyond control of pluripotency, for interchangeable functions of Klf factors during early development. In zebrafish, Klf2a, Klf2b and Klf17 have been shown to regulate ectoderm and mesendoderm development (Gardiner et al., 2005; Kotkamp et al., 2014). In *Xenopus*, inhibition of Klf4 function leads to failure of mesendoderm formation (Cao et al., 2012), and *Klf4* overexpression promotes neuroectoderm and endoderm formation. Interestingly, the expression patterns of KLF2 and KLF17 in human embryos are diametrically opposite to those of Klf2 and Klf17 in mouse (Yan et al., 2013; Blakeley et al., 2015). *klf17* is maternally provided in mouse eggs and its expression abolished around the eight-cell stage, whereas KLF17 becomes significantly upregulated in eight-cell human embryos, following zygotic genome activation (ZGA) (Deng et al., 2014; Yan et al., 2013; Blakeley et al., 2015). Conversely, *klf2* is expressed from the two-cell stage, corresponding to mouse ZGA, and continues through to the blastocyst stage, whereas human *KLF2* is only expressed pre-ZGA (Deng et al., 2014; Yan et al., 2013; Blakeley et al., 2015).

In the current study, we find that loss of either Klf2 or Klf17 activity leads to expanded expression of both neural plate border and neural crest markers, although the increase is more pronounced for the neural plate border markers. This suggests a role for these Klf factors in controlling the extent to which the developing ectoderm retains pluripotency. Consistent with such a role, ectopic expression of either Klf2 or Klf17 inhibits expression of neural crest markers *snai2* and *foxd3*, whereas morpholino depletion of either factor significantly expands expression of *pou5f3.2* at neural plate stages.

We note that a previous study reported that loss of Klf17 (then called Neptune) led to loss rather than to enhanced expression of neural crest markers (S.R. and C.L., unpublished; Kurauchi et al., 2010). Those experiments are not directly comparable to the current study as their MOs were delivered throughout the embryo whereas here they were targeted to the ectoderm. In addition, we have found that partial depletion of Klf17 leads to significantly increased expression of endogenous *klf17* (S.R. and C. L., unpublished), so it is possible that study may have inadvertently been generating a gain-of-function phenotype.

Consistent with a role in inhibiting pluripotency, we found that forced expression of *klf2* or *klf17* prevented blastula explants from transiting to an epidermal, mesodermal or endodermal state. By contrast, depletion of either Klf2 or Klf17 in these explants prolongs the expression of pluripotency factors *sox3* and *pou5f3.2*. A consequence of this is decreased expression of *krt12.4* at stage 13 (Fig. 7C). Strikingly, however, it does not affect expression of the neural plate border factor *pax3*, emphasizing the connectedness of the pluripotency and neural plate border/neural crest GRNs. Given the extended *sox3* and *pou5f3.2* expression, we asked whether Klf depletion would prolong functional pluripotency and found that the time window during which both the neural and endodermal states could be induced was indeed extended.

It is striking that, despite their temporally distinct expression patterns, loss of Klf2 or Klf17 function has similar functional consequences. It seems likely that Klf2 and Klf17 are both required for regulating the exit from pluripotency and thus the ability of cells to transit to lineage-restricted states. Depletion of Klf2 results in more dramatic upregulation of *pouf3.2*, suggesting that its function may be more important at these early stages, and the consequences of its loss secondarily impacts neurula gene expression. Klf17 likely plays a more direct role in regulating neural plate border and neural crest factors, given its striking expression relative to these domains.

Our results suggest that Klf2 and Klf17 are the primary regulators of pluripotent blastula and neural crest stem cells in *Xenopus*, whereas *klf4* is not significantly expressed in these populations. By contrast in amniotes such as mouse and human, Klf4, one of the classical Yamanaka factors, is a key regulator of pluripotency in embryonic stem cells, although Klf2 and Klf17 also play roles. The pluripotency-promoting activities of Klf factors in amniotes contrasts with our findings that, in *Xenopus*, Klf2 and Klf17 restrain pluripotency. Given this divergence, we wished to gain insights into what the ancestral role of these factors might have been in stem vertebrates. Accordingly, we turned to lampreys, one of two extant jawless vertebrates.

Shared features of lamprey and jawed vertebrates likely represent features of their last common ancestor. Lamprey possess *klf17* and *klf2/4* genes; however, only *klf17* is expressed in blastula animal pole cells and neural crest (York et al., 2024). Given these conserved phenotypes, we sought to trace the stepwise evolutionary origins of Klf17 activity in vertebrates by performing molecular phylogenetic analysis and chromosomal synteny comparisons (Fig. S5B,C). Consistent with recent whole-genome analyses in hagfish, lamprey and other vertebrates, our results suggest that a whole-genome duplication event gave rise to *Klf17* and *Klf2/4* in ancestral cyclostomes, features that are in extant lampreys. Importantly, synteny comparisons between *Xenopus* and lamprey show that both *klf2* and *klf4* in *Xenopus* can be mapped to the single lamprey *klf2/4* ortholog (Fig. S5B,C), suggesting that *klf2* and *klf4* arose by tandem gene duplication in stem gnathostomes. These results suggest that the neural crest and pluripotency restraining activities of Klf transcription factors were present in the last common ancestor of jawed and jawless vertebrates, and that they evolved new functions in the lineage leading to extant amniotes. They also support a model in which *klf2*, *klf4* and *klf17* emerged in jawed vertebrates through stepwise evolution via gene duplication of an ancestral *klf2/4/17* gene present in invertebrate chordates.

## MATERIALS AND METHODS

### Embryological methods

Wild-type *Xenopus laevis* embryos were staged and collected in accordance with standard methods (Zahn et al., 2022). *In situ* hybridizations were performed on embryos and explanted animal caps using previously described methods (LaBonne and Bronner-Fraser, 1998). Microinjection of mRNA (Ambion, mMessage mMachine SP6 Transcription Kit) or morpholino (Gene Tools) was carried out in one to four cells at the two- to eight-cell stage as previously described (Lee et al., 2012). Approximately 10-25 ng of translation-blocking morpholinos (Gene Tools) was injected per cell. Manipulated embryos were then cultured in 0.1× Marc's Modified Ringer's Solution (MMR) [0.1 M NaCl, 2 mM KCl, 1 mM MgSO_4_, 2 mM CaCl_2_, 5 mM HEPES (pH 7.8) and 0.1 mM EDTA] until being collected or dissected for animal cap explant assays. All animal cap explants were manually dissected during the early blastula stage and then cultured in 1×MMR until collection. For activin experiments, animal cap explants were dissected and immediately cultured in 1×MMR with 0.1% bovine serum albumin (BSA) and recombinant activin protein (R&D Systems) at a final concentration of 20-40 ng/ml for mesoderm induction and 100 ng/ml for endoderm induction. For neural induction of animal cap explants, BMP signaling activity was inhibited with the small molecule inhibitor K02288 (Sigma) at a final concentration of 20 μM in 1×MMR as previously described (Johnson et al., 2022). Manipulated embryos and/or explants were fixed in 1× MEM [100 mM MOPS (pH 7.4), 2 mM EDTA and 1 mM MgSO_4_] with 4% formaldehyde and dehydrated in methanol prior to *in situ* hybridizations. Results shown are representative of a minimum of three biological replicates.

### DNA constructs

Full-length *Xenopus* Klf2 and Klf17 were obtained from the Xenopus ORFeome (www.xenbase.org/reagents/static/orfeome.jsp) and subcloned into pCS2 vectors for synthesis of mRNA for microinjections. For gain-of-function experiments, each respective mRNA was injected together with mRNA encoding the lineage tracer β-gal. The 3′UTR regions of *klf2* and *klf17* were amplified from genomic DNA isolated from wild-type embryos and subcloned into pGEM-T vector for synthesis of RNA probes. The morpholino antisense oligonucleotides against the 5′UTR-coding regions of *Xenopus klf2* (5′-GAGAATGGTCTCGCTCAGAGCCATC) or *klf17* (5′-GGGTTGAGAAAGCCACACTCATCCT) conjugated to FITC were validated by co-injecting it with an epitope-tagged version of their respective mRNA for western blot analysis.

### Western blot

Five whole embryos were lysed in 1% NP-40 supplemented with protease inhibitors [Complete Mini, EDTA-free tablet (Roche), leupeptin (Roche), aprotinin (Sigma) and phenylmethylsulfonyl fluoride (PMSF; Sigma)]. SDS page and western blot were used to detect proteins. Primary antibodies against c-Myc 9E10 (1:3000; Santa Cruz; sc-40) and actin (1:5000; Sigma; A2066) were used. IRDyes (1:20,000 mouse-800 CW; rabbit-680 TL) and the Odyssey platform (LI-COR Biosciences) were used to detect proteins. Results are representative of a minimum of three biological replicates.

### RNA isolation, cDNA synthesis and qRT-PCR

RNA was isolated from uninfected control or manipulate animal cap explants (15-20 explants) using Trizol (Life Technologies). 1 μg of purified RNA was used as a template for synthesizing cDNA using a High Capacity Reverse Transcription Kit (Life Technologies). Quantitative qRT-PCR was performed using SYBR Premix (Clonetech, RR820W). Expression was normalized to ornithine decarboxylase (ODC) and the fold change was calculated using the ΔΔCT method. The results show the mean of at least three independent biological replicates±s.e.m. An unpaired, two-tailed *t*-test was used to determine significance. Primers used and their sequences are listed in Table S1.

### Hybridization chain reaction

Hybridization chain reaction (HCR) methodologies are adapted from (Choi et al., 2018). Whole embryos were hybridized with DNA probe sets for *klf17*, *pax3*, *snai2*, *foxd3*, *zic1*, *sox2* and *pou5f3.2.* (Molecular Instruments) and incubated overnight at 37°C. Probe was removed, samples washed and then incubated overnight with DNA hairpins labeled with Alexa 647 or Alexa 546 (Molecular Instruments). Unbound hairpins were removed via 5× SSCT washes followed by PBS washes and incubated in DAPI (1:5000; Life Technologies). Samples were mounted and imaged using a Nikon C2 upright confocal with two GaAsP detectors and four standard laser lines with either 4× or 10× objectives.

### Line profile analysis

Line profile measurements of *klf17*, *pax3* and *snai2* were made on multi-channel maximum intensity projections from confocal files using the python packages scikit-image (Van Der Walt et al., 2014). Intensity profiles for each fluorescent channel were measured using the profile_line function from scikit-image along a user defined line with endpoints chosen manually using the python package, mpl_point_clicker (https://github.com/ianhi/mpl-point-clicker). The intensity profile was then normalized to a range between 0 and 1 by subtracting the minimum intensity value and dividing by the range (maximum-minimum intensity) for each channel. In order to compare multiple line profiles from each image, the average *pax3* or *snai2* intensities were first fit to an exponential curve and the peaks of each centered at zero. After curve fitting, the common x-values (pixel distance) were identified by calculating the intersection of the x-values across all measurement curves and trimmed to only include common x-values. The mean and standard error of the normalized intensities of *klf17* in combination with either *pax3* or *snai2* were then calculated and plotted across the x-values.

### Multi sequence alignment

Multiple sequence alignment (MSA) of *Xenopus laevis* Klf2 (NP_001080430.1), Klf4 (XP_041436022) and Klf17 (NP_001082133) protein sequences, obtained from Xenbase, was performed using MUSCLE (EBI) with the EMBL-EPI Job Dispatcher sequence analysis tool. The resulting alignment was visualized and pairwise percent identify calculations were carried out using Jalview.

### Phylogenetic analysis and synteny of Klf transcription factors

Full-length Klf-family proteins were downloaded from NCBI. The sequences were aligned using MAFFT (v7.490 with <—maxiterate 1000 —globalpair>, and trimmed using trimAl (v1.4.1). The <automated1> option was chosen to automatically determine the optimal method for trimming. The trimmed alignment file was converted to NEXUS format for phylogenetic analysis in MrBayes (v3.2.7a). We used the following parameters: <prset aamodel=mixed>, <mcmcp ngen=500,000>; mcmcp nchains=4; mcmcp samplefreq=100; *Drosophila* Krüppel was specified as outgroup. Consensus trees were visualized using iTOL (https://itol.embl.de). Synteny analysis was performed by comparing the coding sequences of *Xenopus* genes surrounding the *klf2*, *klf4* and *klf17* loci to those surrounding the *klf2/4* and *klf17* loci in the germline genome assembly of lamprey.

### Animals

All animal procedures were approved by the Institutional Animal Care and Use Committee, Northwestern University, and were carried out in accordance with the National Institutes of Health's Guide for the Care and Use of Laboratory Animals.

## Supplementary Material

10.1242/develop.204634_sup1 Supplementary information
