# Supplementary material for: Krüppel-like factors play essential roles in regulating pluripotency and the formation of neural crest stem cells
Source: Development. 2025 May 1;152(9):dev204634. doi: 10.1242/dev.204634 (PMC12070069; doi:10.1242/dev.204634)
Supplement: Supplementary information [file develop-152-204634-s1.pdf]

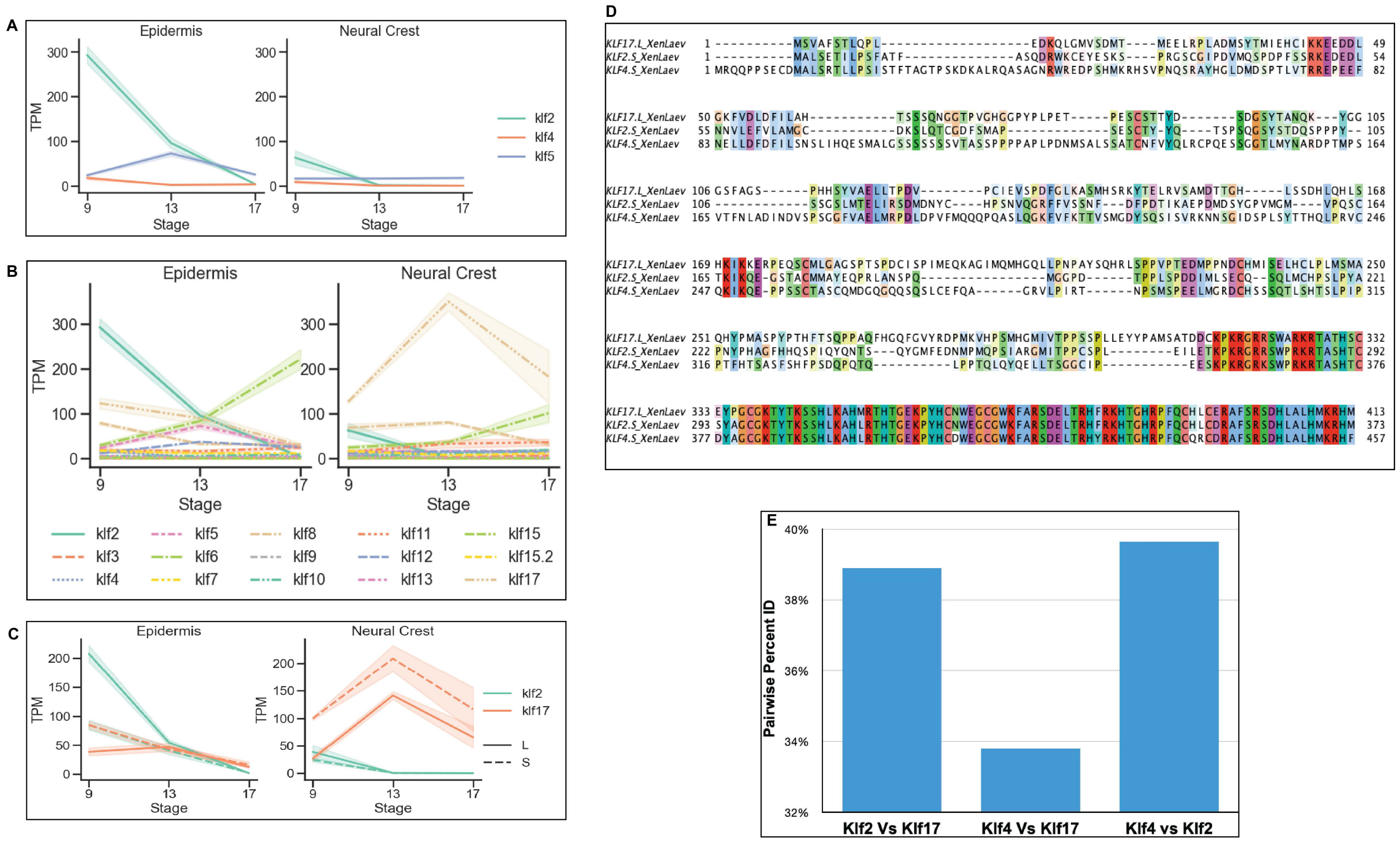

Fig. S1. TPM plots of klf factors in epidermal and neural crest explants. (A-B) Plots of the average TPMs of Klf factors in epidermis and neural crest explants at blastula (stage 9), early neurula (stage 13), and late neurula (stage 17) stages. Shading represents the standard error of mean. (A) Plots of klf2, klf4 and klf5 TPMs. (B) Plots of all 15 klfs in the Xenopus laevis genome. (C) Plots of Klf2 and Klf17 L & S alleles (D) Multi-Sequence Alignments of human, mouse, and Xenopus Klf2, Klf4, & Klf17 (D) Mult-Sequence Alignments Xenopus Klf2, Klf4, and Klf17. (E) Plot comparing percent identies calculated from pairwise alignments Between Klf2, Klf4, and Klf17.

Development • Supplementary information

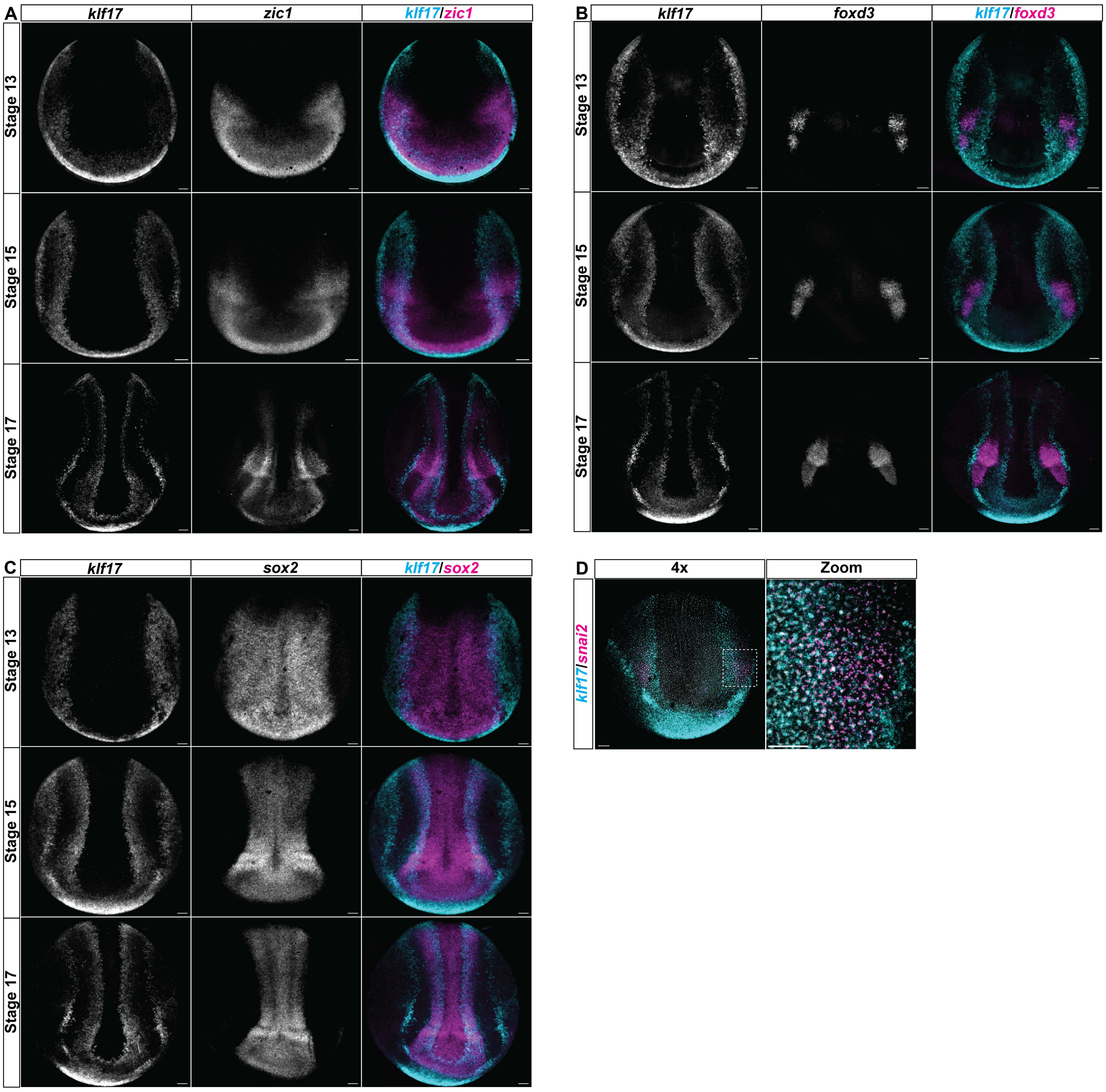

Fig. S2. Additonal HCR of overlapping expression patterns of Klf17 with neural plate, neural plate border and neural crest changes during neurulation. (A-C)Whole embryos probed with HCR oligos examining the expression patterns of (A) klf17 (cyan) and the neural crest marker foxd3 (magenta) ; (B) klf17 (cyan) and theneural plate border marker zic1 (magenta); or (C) klf17 (cyan) and the neural plate marker sox2 (magenta) at early (stage 13), mid (stage 15), and late (stage 17) neurulation. Scale bars: 150 µm. (D) Zoom of 4x HCR images from Fig. 2C showing co expression of klf17 with the neural crest marker snai2 at stage 13. Dashed box: zoom area; Scale bars: 150µm (4x) or 50µm (zoom).

Development • Supplementary information

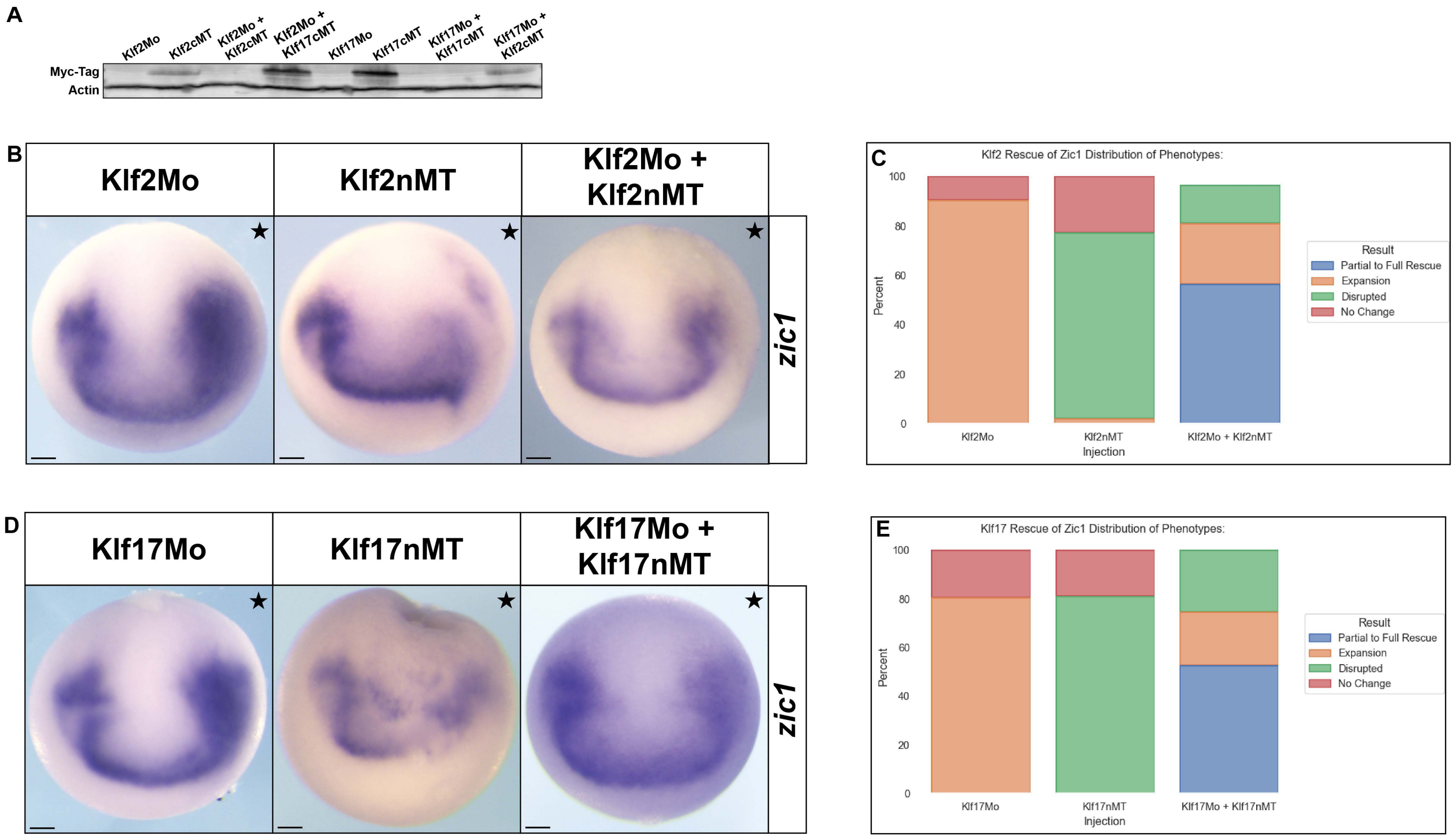

Fig. S3. Klf2 and Klf17 Morpholino Validation. (A) Western blot validation of Klf2 and Klf17 morpholinos. Lanes 3 & 7 show each morpholino blocks the translation of their respective Klf. Lanes 4 & 8 show each morpholino does not block translation of the other Klf. (B) In situ hybridizations showing the rescue of zic1 expression in klf2-morphant embryos co-injected with epitope tagged klf2 mRNA (C) Stacked bar graphs of the percent distribution of phenotypes for the klf2 rescue of zic1 expression (Klf2Mo n=52; klf2-nMT(n=92; Klf2Mo + klf2-nMT n= 90). (D) In situ hybridizations showing the rescue of zic1 in klf17-morphant embryos co-injected with epitope tagged klf17 mRNA. (E) Stacked bar graphs of the percent distribution of phenotypes for the klf17 rescue of zic1 expression (Klf17Mo n=62; klf17-nMT n=63; Klf17Mo + klf17-nMT n=55) . cMT, C-terminal myc tag; nMT, N-terminal myc tag; MO, morpholino.

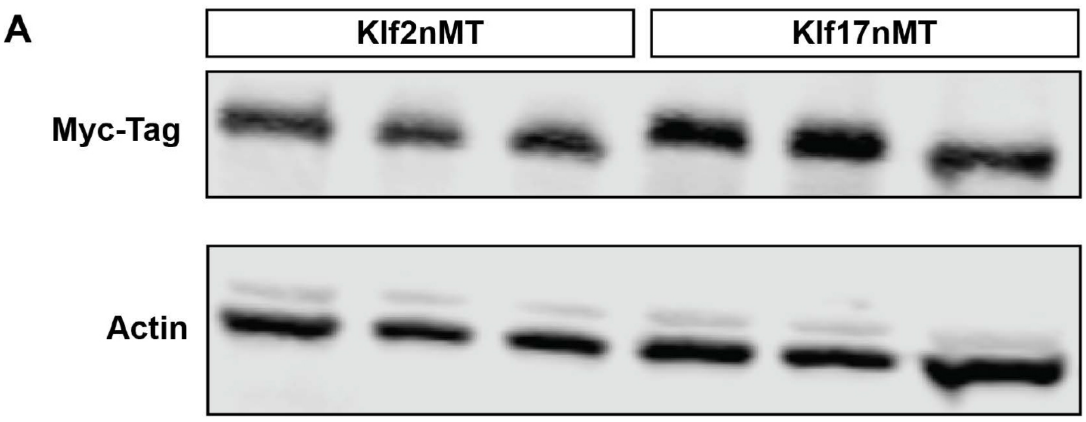

Fig. S4. Western Blot to Validate Protein Levels. (A) Western blot of three replicates of epitope tagged *klf2* and *klf17* used in gain of function experiments at consistent levels. nMT, N-terminal myc tag.

Development • Supplementary information

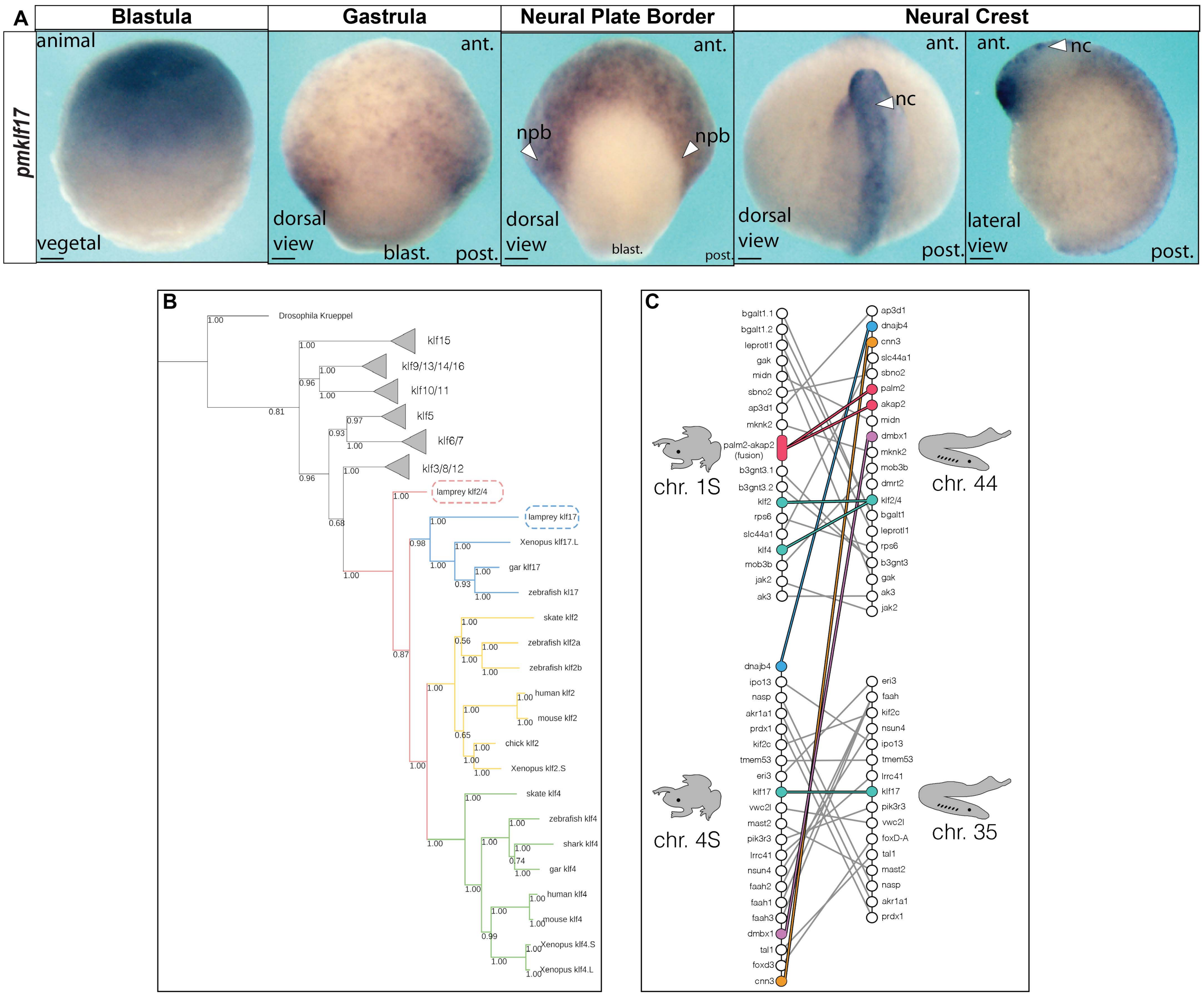

Fig. S5. Evolution and Conservation of Klf factors. (A) In situ hybridizations of *klf17* expression in the blastula, gastrula, neural plate border and neural crest of wild type sea lamprey embryos. (B) Phylogenetic tree of Klf factors across several species. (C) Synteny analysis of klf2 and klf17 in Petromyzon marinus (sea lamprey) and Xenopus laevis. pm, Petromyzon marinus ; ant, anterior; post., posterior; blast., blastopore; npb, neural plate border; nc, neural crest

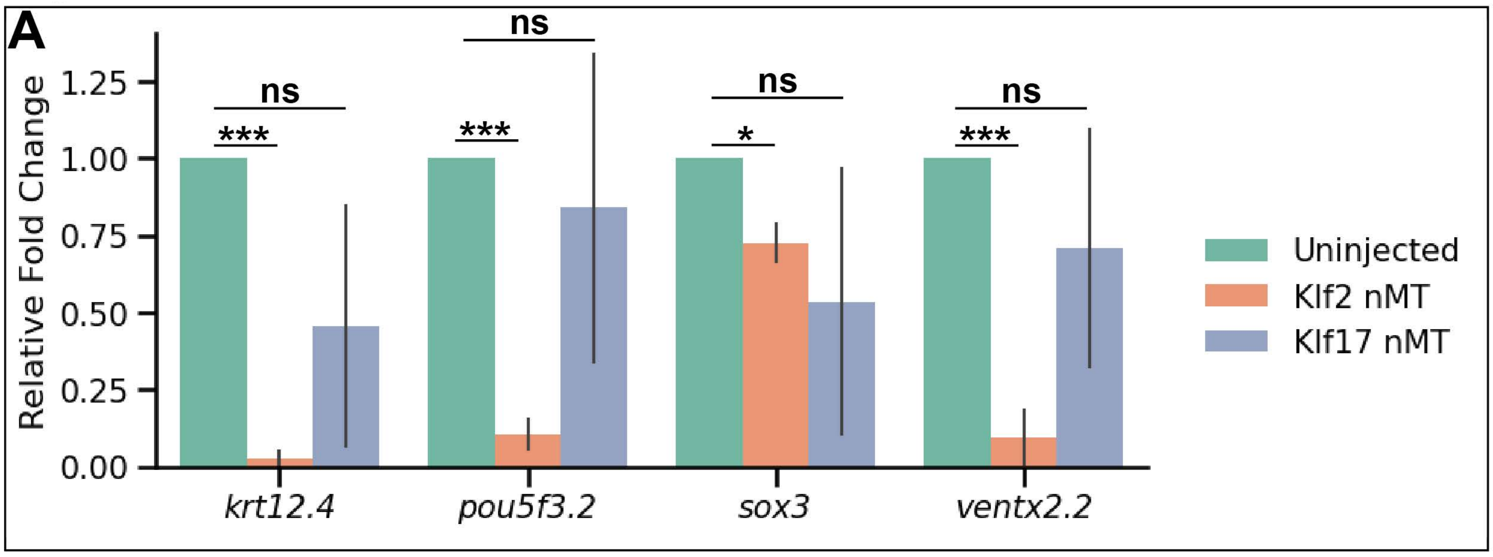

Fig. S6. qPCR of Explants with Ectopic Klf2 and Klf17(A) Plots of relative fold change of uninjected, ectopic Klf2 or ectopic Klf17 explants from qPCR. Normalized to uninjected explants (stage13) for *krt12.4, pou5f3.2*, *sox3*, and *ventx2.2*. *p ≤ 0.05; ***p ≤ 0.001, ns not signficant; error bars: standard error of the mean

Development • Supplementary information

Table S1. qPCR primers

Gene	Forward	Reverse
ODC	TGAAAACATGGGTGCCTACA	TGCCAGTGTGGTCTTGACAT
sox3	CACAACTCGGAGATCAGCAA	TCGTCGATGAAGGGTCTTTT
pou5f3.2	AATGGGGCAATTAATGAACG	GGGAACCTCCTCCTCATTGT
ventx2.2	GCTACACAGGGACACAACCTC	GCCTGAGTCAGTGCTAGTGC
krt12.4	TTGGTGCTGGGTCTAAAGATT	TTTGCAGAGTCACTGTAGCATT
